# Fundamental hemodynamic mechanisms mediating the response to myocardial ischemia in conscious paraplegic mice: cardiac output versus peripheral resistance

**DOI:** 10.14814/phy2.13214

**Published:** 2017-03-29

**Authors:** Heidi L. Lujan, Stephen E. DiCarlo

**Affiliations:** ^1^ Department of PhysiologyWayne State University School of MedicineDetroitMichigan

**Keywords:** Coronary artery disease, myocardial ischemia, spinal reflex

## Abstract

Autonomic dysfunction, a relative sedentary lifestyle, a reduced muscle mass and increased adiposity leads to metabolic abnormalities that accelerate the development of coronary artery disease (CAD) in individuals living with spinal cord injury (SCI). An untoward cardiac incident is related to the degree of CAD, suggesting that the occurrence of a significant cardiac event is significantly higher for individuals with SCI. Thus, understanding the fundamental hemodynamic mechanisms mediating the response to myocardial ischemia has the potential to positively impact individuals and families living with SCI. Accordingly, we systematically investigated if thoracic level 5 spinal cord transection (T_5_X; paraplegia) alters the arterial blood pressure response to coronary artery occlusion and if the different arterial blood pressure responses to coronary artery occlusion between intact and paraplegic mice are mediated by changes in cardiac output and or systemic peripheral resistance and whether differences in cardiac output are caused by changes in heart rate and or stroke volume. To achieve this goal, the tolerance to 3 min of coronary artery occlusion was determined in conscious intact and paraplegic mice. Paraplegic mice had an impaired ability to maintain arterial blood pressure during coronary artery occlusion as arterial pressure fell to near lethal levels by 1.38 ± 0.64 min. The lower arterial pressure was mediated by a lower cardiac output as systemic peripheral resistance was elevated in paraplegic mice. The lower cardiac output was mediated by a reduced heart rate and stroke volume. These results indicate that in paraplegic mice, the arterial pressure response to coronary artery occlusion is hemodynamically mediated primarily by cardiac output which is determined by heart rate and stroke volume.

## Introduction

Individuals living with spinal cord injury (SCI) have a significantly greater risk for heart disease and stroke, and cardiovascular disease is the leading cause of death and morbidity (Kessler et al. 1986; Cardus et al. 1992; Whiteneck et al. 1992; DeVivo et al. 1993; DeVivo 1997; Frankel et al. 1998; Groah et al. 2001; West et al. 2012; Cragg et al. 2013). The risk for cardiovascular disease is associated with an unstable autonomic control of the heart and vasculature, a relatively sedentary lifestyle (Inskip et al. 2010) and blood lipid profiles consisting of elevated total and low‐density lipoprotein cholesterol and depressed high‐density lipoprotein cholesterol (Bauman et al. 1992, 1999; Cardus et al. 1992). Relative inactivity associated with SCI also results in a reduced muscle mass and increased adiposity. As such, individuals living with SCI often experience insulin resistance, hyperinsulinemia and an atherogenic profile that contributes to early development of coronary artery disease (CAD). Specifically, autonomic dysfunction, relative inactivity and adverse changes in body composition lead to metabolic changes that promote CAD in individuals living with SCI (Whiteneck et al. 1992; Bauman et al. 1993, 1994; Lee et al. 2006; Bauman and Spungen 2007; Orakzai et al. 2007). An untoward cardiac incident is related to the degree of CAD, suggesting that the occurrence of a significant cardiac event is significantly higher for individuals with SCI (Fleg et al. 1990). Accordingly, understanding the response to myocardial ischemia has the potential to positively impact individuals and families living with SCI and provides an improved approach to clinical care.

In this context, the cardiovascular response to coronary artery occlusion is mediated, in part, by a cardiac spinal reflex as well as the arterial baroreflex. Both reflexes increase heart rate and arterial pressure during reductions in cardiac output caused by the myocardial ischemia‐induced reductions in cardiac performance. Specifically, ischemic metabolites are released during myocardial ischemia that stimulate cardiac spinal afferents (Uchida and Murao 1974; Baker et al. 1980; Malliani et al. 1981; Pan et al. 1999; Fu and Longhurst 2002, 2005; Tjen et al. 2002; Fu et al. 2005) leading to elevations in sympathetic activity, heart rate, and arterial blood pressure (Malliani et al. 1981; Meller and Gebhart 1992; Longhurst et al. 2001). Transection of the dorsal roots disrupts this excitatory cardiac sympathetic reflex and reduces heart rate and arterial pressure during myocardial ischemia (Lujan et al. 2011). In addition, cardiac output and arterial pressure are reduced during myocardial ischemia which initiates a dramatic arterial baroreflex‐mediated increase in sympathetic outflow and decrease in parasympathetic outflow to the heart as well as an increased sympathetic outflow to the vasculature. Importantly, individuals with SCI at thoracic level 5 have a functional loss of the cardiac spinal reflex and the arterial baroreceptor reflex *below* the level of the injury and thus fail to excite sympathetic preganglionic neurons below T_5_. Accordingly, the tolerance to coronary artery occlusion is expected to be markedly impaired in T_5_X mice (Lujan et al. 2011).

Accordingly, we systematically investigated if thoracic level 5 spinal cord transection (T_5_X; paraplegia) alters the arterial blood pressure response to coronary artery occlusion and if the different arterial blood pressure responses to coronary artery occlusion between intact and paraplegic mice are mediated by changes in cardiac output and or peripheral resistance and whether differences in cardiac output are mediated by variations in heart rate and or stroke volume. To achieve this goal, mice were instrumented to record cardiac output, arterial pressure, and the electrocardiogram (ECG) (Lujan et al. 2012a; Lujan and DiCarlo 2013a, 2014c). In addition, a vascular occluder was placed around the left main coronary artery (Lujan et al. 2012a; Lujan and DiCarlo 2013a, 2014c). Following recovery, mice were subjected to T_5_X or sham T_5_X. Two weeks later, the tolerance to 3 min of occlusion of the left main coronary artery was determined by using the coronary vascular occluder.

## Materials and Methods

### Animals

All procedures involving animals were in accord with The American Physiological Society's Guiding Principles in the Care and Use of Animals. Approval for all procedures and protocols involving animals was provided by the Animal Care and Use Committee of Wayne State University. Twelve adult male C57BL/6 mice 3–4 months of age, (*n *=* *6, midthoracic spinal cord transected [T_5_X]; and *n *=* *6, sham transected [intact]) were studied 14 days postspinal cord transection or sham transection.

The study was designed to determine the fundamental hemodynamic mechanisms responsible for the impaired capacity to maintain arterial blood pressure during coronary artery occlusion in conscious paraplegic (T_5_X) mice. Although intact mice tolerate coronary artery occlusion for at least 90 min leading to myocardial infarction (Lujan et al. 2012a); paraplegic mice tolerate coronary artery occlusion for <2 min. Accordingly, a 3‐min period of occlusion was selected for comparison between groups.

Following anesthesia with pentobarbital sodium (60 mg/kg ip), the mice were atropinized (0.05 mg/kg ip), intubated, and prepared for aseptic surgery. Supplemental doses of pentobarbital sodium (10–20 mg/kg ip) were given if the mice responded to tail pinch.

### Thoracotomy procedures

Following anesthesia, a thoracotomy was created through the second intercostal space and the sheath of the pericardium that spreads over the ascending aorta was removed. An appropriately sized (1.6‐mm) silicone‐type Doppler ultrasonic flow probe (Iowa Doppler Products, Iowa City, IA) was placed around the ascending aorta as recently described (Lujan and DiCarlo 2013a, 2014c). The flow probe wires were channeled subcutaneously and exited at the dorsal aspect of the neck. Next, a vascular occluder was positioned around the coronary artery as recently described (Lujan et al. 2012a; Lujan and DiCarlo 2013a, 2014c). The ends of the vascular occluder were passed through guide tubing and exited at the dorsal aspect of the neck (Lujan et al. 2012a; Lujan and DiCarlo 2013a, 2014a,c). The local anesthetic, bupivicaine, was injected (sq) at the incision site. Next, ECG electrodes (DataSciences International, Standard Lead Coupler Kit: 276‐0031‐001) were placed in a modified lead II configuration, tunneled subcutaneously and exited at the dorsal aspect of the neck as previously described in mice (Lujan et al. 2012a; Lujan and DiCarlo 2013a, 2014a,c). Finally, a catheter from a telemetry device (Data Sciences International, PA‐C10), for recording arterial pressure, was inserted into the left carotid artery until the tip reached the aortic arch (Kurtz et al. 2014). The transmitter body was placed subcutaneously on the left side.

All animals remained ventilated and on a feedback‐based temperature control system until recovered from the anesthesia. Following recovery from the anesthesia, the mice were housed in a “rodent recovery cage” (Thermocare^®^ Intensive Care Unit, Braintree Scientific, Braintree MA) and returned to the animal facility the following day. Carprofen (5 mg/kg, sq) and cefazolin (10 mg/kg, sq) were continued for 2 days. A minimum ten‐day recovery period was enforced during which the mice were weighed and acclimatized to the laboratory and investigators.

### Spinal cord transection (T_5_X) or Sham transection

Following the minimum ten‐day recovery period, the mice were anesthetized as defined earlier and subjected to complete thoracic level 5 spinal cord transection (T_5_X) or sham spinal cord transection. Specifically, the spinous process and laminae of the 4th thoracic vertebrae were removed exposing the 5th thoracic spinal cord segment. The 5th thoracic spinal cord segment is positioned below the 4th thoracic vertebral because the spinal cord is shorter than the vertebral column. Sympathetic preganglionic neurons projecting to the heart leave the spinal cord via the 1st through 4th thoracic spinal cord segments (Strack et al. 1988). Accordingly, spinal cord transection between the 5th and 6th thoracic spinal cord segments maintains supraspinal control of preganglionic neurons projecting to the heart but loss of supraspinal control of preganglionic neurons below the thoracic level 5 spinal cord segment. The extensiveness of the spinal cord transection was established by visually inspecting the transection site. Undistinguishable procedures were followed for the sham transected mice, except the spinal cord was not transected. All mice received a supplemented nutritious diets (Bio‐Serv, Frenchtown, NJ).

### Experimental procedures

#### Response to myocardial ischemia

The experimental procedures were conducted in conscious and unrestrained mice. All physiological measurements (cardiac output, arterial blood pressure, and the electrocardiogram) were made as previously described (Lujan et al. 2012a; Lujan and DiCarlo 2013a, 2014a,c; Kurtz et al. 2014) with the mice in their standard home cages during the light cycle. Temperature within the cage was maintained within the thermoneutral zone for mice of approximately 29–31°C (Swoap et al. 2004). Mice adapted to the experimental conditions for approximately 2 h to ensure stable hemodynamic conditions.

**Figure 1:**
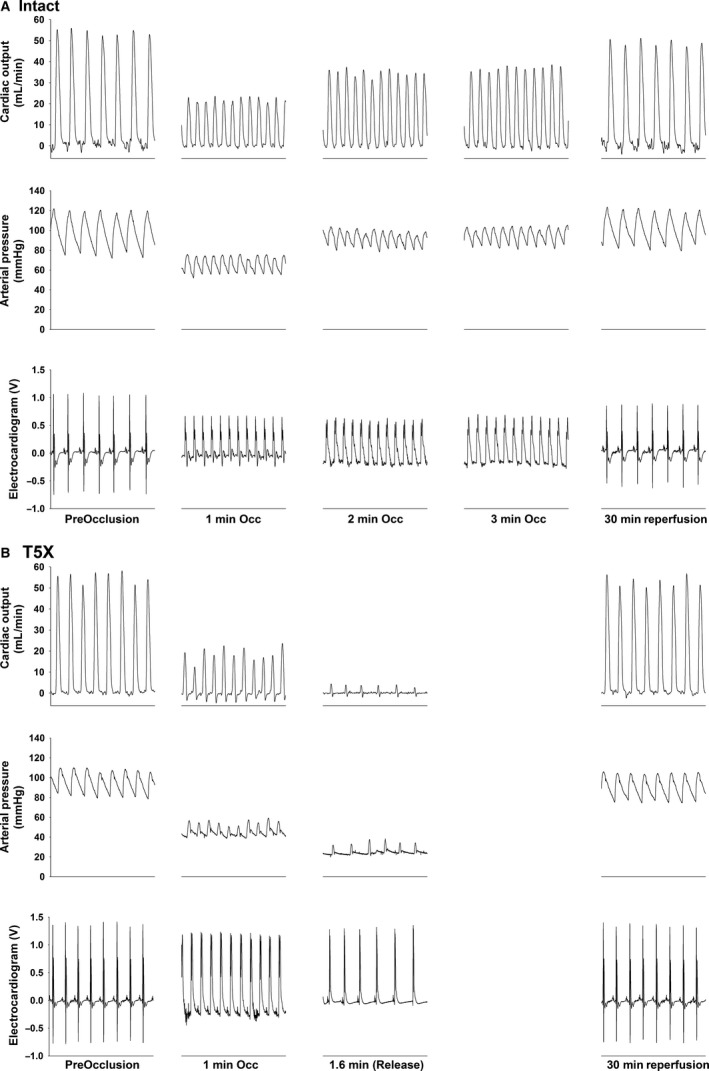
One second original recordings of cardiac output, arterial pressure, and the electrocardiogram for one intact (panel A) and one paraplegic (panel B) mouse are presented. Coronary artery occlusion significantly reduced arterial pressure in paraplegic mice. The reduced arterial pressure was mediated by a reduced cardiac output. None of the T_5_X mice tolerated 3 min of occlusion. In this example, the duration of the occlusion for the T_5_X mouse was 1.6 min.

Following the adaptation period, the left main coronary artery was temporarily occluded for 3 min using the coronary vascular occluder as previously described (Lujan et al. 2012a; Lujan and DiCarlo 2013a, 2014a,c). Expected responses in the ECG (peaked T wave and S‐T segment elevation), and a rapid fall in cardiac output and arterial pressure documented coronary artery occlusion (Fig. 1) (Lujan et al. 2012a; Lujan and DiCarlo 2013a, 2014a,c). Upon release, *intact* mice experienced sustained ventricular tachycardia (absence of p wave, wide QRS complex) >900 beats/min as well as a reduced cardiac output and arterial blood pressure (Curtis et al. 2013). Gentle chest compressions or, in some cases, person to mouse ventilation via a small tube over the nose and mouth restored normal sinus rhythm.

None of the paraplegic mice tolerated the occlusion for more than 2 min. The occlusion was terminated when cardiac output and arterial pressure were virtually zero and the mice required gentle compression of the thorax and person to mouse ventilation using a customized tube over the nose and mouth for survival. One week after the experimental protocol, the hearts were harvested from all mice.

#### Determination of the ischemic zone (*n* = 3/group)

One week after completion of the experimental protocols, the mice were deeply anesthetized and the hearts and aortae were removed with the coronary vascular occluder in place. The hearts and aortae were immediately immersed in heparinized saline to remove remaining intracavitary blood. Subsequently, the aorta was cannulated and perfused with 1–2 mL saline until the saline drained clear from the coronary sinus (Bohl et al. 2009). Next, the coronary vascular occluder was tied to block flow through the left main coronary artery. Evans blue dye (250 *μ*L, 0.5%) was then sent via the aorta, so that the dye entered and stained the nonischemic zone of the heart. The ischemic zone remained unstained. The heart was then trimmed leaving only the ventricles. The ventricles were rinsed to remove the excess dye; then sliced from apex to base into approximately four to six sections (∼1 mm thickness). Ventricular sections were compressed between two microscope slides to achieve uniform thickness and images of each ventricular section were obtained with a digital camera mounted on a microscope. The total ventricular section area and the ischemic ventricular zone of each heart section were quantified using the SPOT Imaging software (version 4.7, SPOT Imaging Solutions, Sterling Heights, MI). As noted above, the ischemic ventricular zone was devoid of Evans blue dye. The percentage of the ischemic ventricular zone was calculated for each section by dividing the ischemic ventricular zone by the total ventricular area. The ischemic zones for all ventricular sections were averaged to obtain the percentage of the ventricle that was ischemic. There were no differences in the ischemic ventricular zone between the intact and paraplegic mice (62 ± 5 vs. 60 ± 4%).

##### Preparation of heart sections (*n* = 3/group)

One week after completion of the experimental protocols, the mice were deeply anesthetized and the hearts and aortae were removed (Lujan et al. 2012a; Lujan and DiCarlo 2013a, 2014c). The ventricles were quickly rinsed in 10 mmol/L Tris, 0.9% NaCl, 0.05% thimerosal in 10 mmol/L phosphate buffer, pH 7.4 (TPBS), then immersion fixed in formaldehyde/zinc fixative for 60 min, washed in TPBS (3 × 10 min), then cryoprotected overnight in 30% sucrose (prepared in half strength TPBS). The OCT embedded ventricles were sectioned transversely from the apex to the base (short axis) at 10 *μ* intervals leaving an interval of 300 *μ* between ventricular sections. Ventricular sections were thaw mounted on Superfrost Plus slides and stained with Masson Trichrome to determine if the vascular occlusion caused cardiac damage.

### Data analysis

All physiological recordings were obtained at a sampling frequency of 4 kHz. All data were expressed as means ± SE. A two‐way repeated measures ANOVA was used to determine time and group (intact and T5X) responses in arterial pressure, cardiac output, systemic peripheral resistance, heart rate and stroke volume before coronary artery occlusion, during coronary artery occlusion and 30 min postocclusion.

A second two‐way repeated measures ANOVA was used to compare the change in all hemodynamic variables from preocclusion values during coronary artery occlusion and 30 min postocclusion. Finally, a one‐way repeated measures ANOVA was used to compare all hemodynamic variables from preocclusion values, during coronary artery occlusion and 30 min postocclusion in intact and T5X groups. The Holm‐Sidak post hoc procedure was used for post hoc pair wise comparisons. A value of *P *<* *0.05 was considered statistically significant.

### Standard calculations

Cardiac output (Q), in mL/min, was calculated by:

Q=kHZ of Doppler frequency shift×K

where *K* = 1.24 × *d* ^2^, [*d* is the cuff diameter of the flow probe, (Haywood et al. 1981)].

Systemic peripheral resistance was calculated by:

$$\begin{array}{c} \text{Peripheral resistance (mmHg/mL/min)}=\text{mean arterial}\\ \text{pressure (mmHg)}/\text{cardiac output (mL/min)} \end{array}$$

Stroke volume (SV), in *μ*L/beat, was calculated by:

$$\begin{array}{cc} \text{SV}(\mu \text{L/beat}) & =\text{Cardiac Output}(\mu L/\min)/\\ & \text{heart rate}(\text{beats}/\min) \end{array}$$

## Results

Original recordings of cardiac output, arterial blood pressure, and the ECG before coronary artery occlusion (preocclusion) at minutes 1, 2, and 3 of coronary artery occlusion and following 30 min of reperfusion in a conscious intact (Panel A) and paraplegic (Panel B) male mouse are presented in Figure 1. Coronary artery occlusion significantly reduced arterial pressure only in the mouse with paraplegia (Panel B). The ECG showed the expected changes including a peaked T wave and S‐T segment elevation. None of the T_5_X mice tolerated 3 min of coronary artery occlusion and all mice required resuscitation at the time of release. The duration of occlusion before requiring intervention in this example was 1.6 min for the paraplegic mouse.

**Figure 2:**
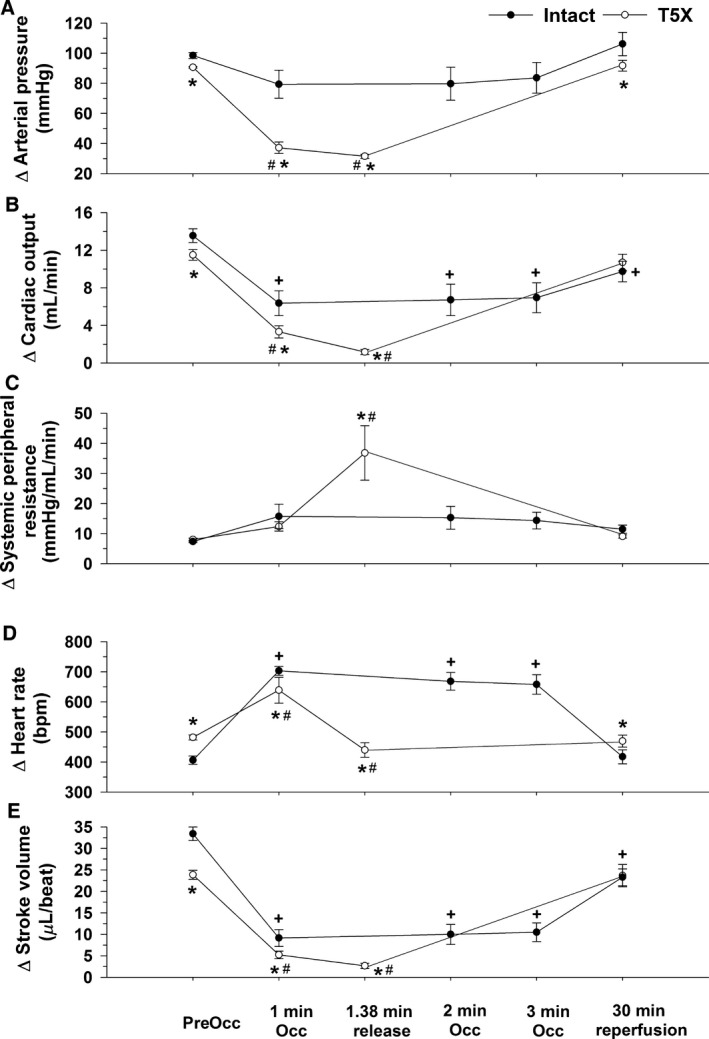
Arterial pressure (Panel A), cardiac output (Panel B), systemic peripheral resistance (Panel C), heart rate (Panel D), and stroke volume (Panel E) in conscious intact and paraplegic mice are presented. Preocclusion arterial pressure, cardiac output, and stroke volume were significantly lower while heart rate was significantly higher in T_5_X mice. There was no difference in preocclusion systemic peripheral resistance. The lower preocclusion arterial pressure in the mice with paraplegia was the result of a lower cardiac output with no contribution of systemic peripheral resistance. Since preocclusion heart rate was significantly elevated in the mice with paraplegia, the lower preocclusion cardiac output was the result of a lower stroke volume. Moreover, T_5_X mice had an impaired ability to maintain arterial blood pressure during coronary artery occlusion as arterial pressure fell to near lethal levels by 1.38 ± 0.64 min. The impaired ability to maintain arterial pressure during coronary artery occlusion in paraplegic mice was mediated by a lower cardiac output as peripheral resistance was elevated. The lower cardiac output was mediated by a reduced stroke volume and heart rate. *P ≤ 0.05, T5X versus Intact #*P* ≤ 0.05, T5X; Preocclusion versus 1 min, 1.38 min release, 30 min reperfusion +*P* ≤ 0.05, Intact; Preocclusion versus 1 min, 2 min, 3 min, 30 min reperfusion

Figure 2 presents arterial blood pressure (Panel A), cardiac output (Panel B), systemic peripheral resistance (Panel C), heart rate (Panel D), and stroke volume (Panel E) before coronary artery occlusion (preocclusion), during coronary artery occlusion and following 30 min of reperfusion in conscious intact and paraplegic mice. Preocclusion arterial pressure, cardiac output, and stroke volume were significantly lower while preocclusion heart rate was significantly higher in the T_5_X mice. There was no difference in preocclusion systemic peripheral resistance.

The lower preocclusion arterial pressure (Panel A) in the mice with paraplegia was the result of a lower cardiac output (Panel B) with no contribution of systemic peripheral resistance (Panel C). Moreover, since preocclusion heart rate was significantly elevated in the mice with paraplegia (Panel D), the lower preocclusion cardiac output was the result of a lower stroke volume (Panel E).

Furthermore, acute coronary artery occlusion significantly reduced arterial blood pressure only in the paraplegic mice (Fig. 2, Panel A). Thus, paraplegic mice had an impaired ability to maintain arterial blood pressure during coronary artery occlusion as arterial pressure fell to near lethal levels by 1.38 ± 0.64 min. In contrast, intact mice tolerated the ischemic insult, without a significant reduction in arterial pressure, for the entire 3 min without incidence (Fig. 2, Panel A).

**Figure 3:**
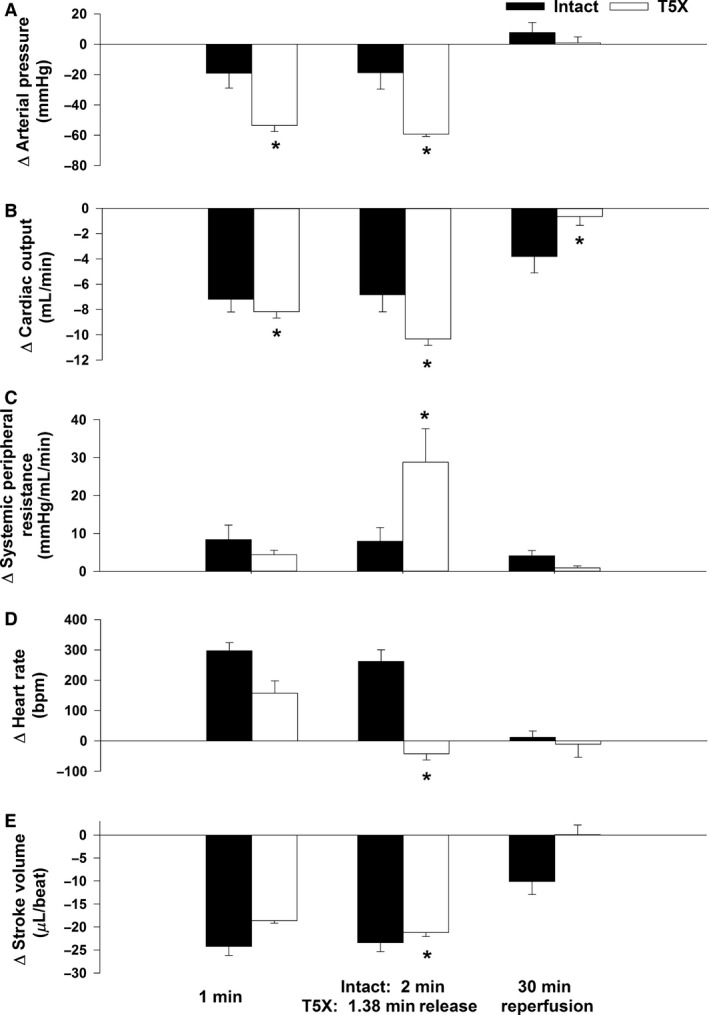
Changes in arterial pressure (Panel A), cardiac output (Panel B), systemic peripheral resistance (Panel C), heart rate (Panel D), and stroke volume (Panel E) from control in conscious intact and paraplegic mice are presented. Paraplegic mice had an impaired ability to maintain arterial blood pressure during coronary artery occlusion as the change in arterial pressure was greater in paraplegic mice (Panel A). The lower arterial pressure was mediated by a larger change in cardiac output (Panel B) as systemic peripheral resistance was elevated in paraplegic mice (Panel C). The lower cardiac output was mediated by a reduced heart rate (Panel D). *P ≤ 0.05, T5X versus Intact

The impaired ability to maintain arterial blood pressure during coronary artery occlusion in paraplegic mice was mediated by a lower cardiac output. Specifically, the reduction in cardiac output was greater in the paraplegic mice at minute 1 of occlusion and at the time of release compared with the 2‐min time point in the intact mice (Fig. 2, Panel B and Fig. 3, Panel B). Correspondingly, the increase in peripheral resistance was greater in the paraplegic mice at the time of release compared with the 2‐min time point in the intact mice (Fig. 2, Panel C and Fig. 3, Panel C). The lower cardiac output was mediated by a reduced heart rate and stroke volume as the increase in heart rate was smaller in the paraplegic mice (Fig. 2, Panel D and Fig. 3, Panel D) while stroke volume was lower in the paraplegic mice (Fig. 2, Panel E).

Cardiac output and arterial pressure returned near preocclusion values within 30 min of reperfusion suggesting that little or no tissue damage occurred during the ischemia.

**Figure 4:**
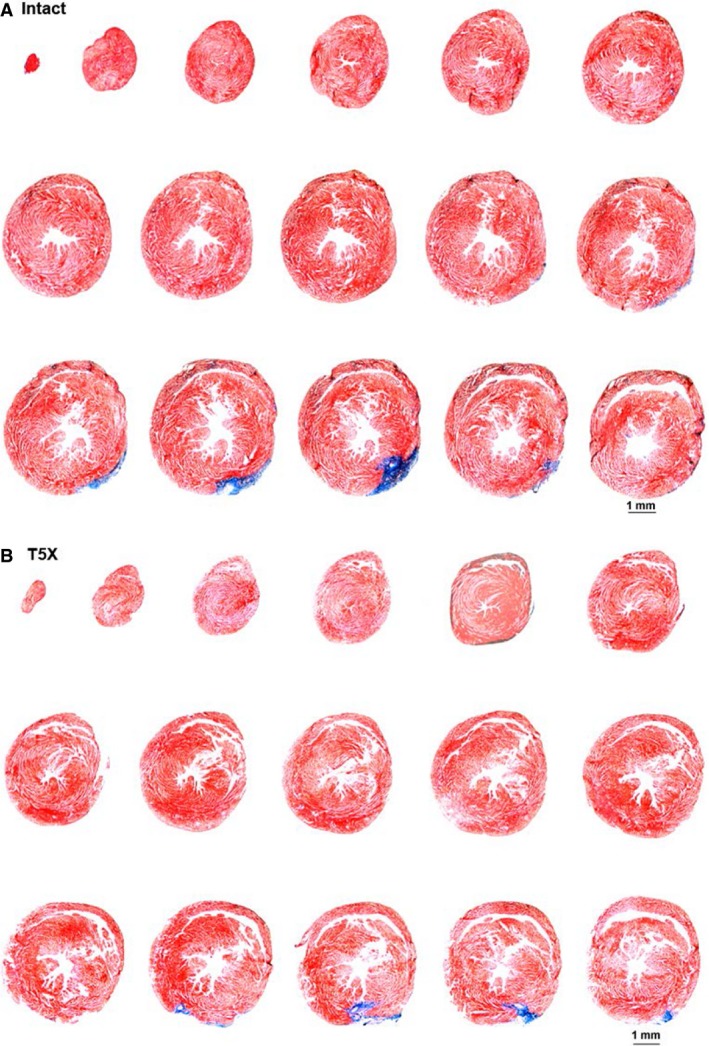
Ventricular sections from one intact and paraplegic mouse are shown. The only collagen (i.e., blue stain, documenting tissue injury) shows placement of the coronary artery occluder. Thus, little tissue damage occurs during the ischemia.

Photomicrographs of ventricular sections from intact and paraplegic mice are presented in Figure 4. The 10 *μ* sections were obtained from the apex through the base (short axis) at 300 micron intervals and processed with Masson Trichrome stain. The only collagen (i.e., blue stain, documenting tissue injury) shows placement of the coronary artery occluder. Thus, little tissue damage occurred during the ischemia and reperfusion protocol. Furthermore, no differences in the ischemic zone between the intact and paraplegic mice were observed (62 ± 5 vs. 60 ± 4%) documenting that the mice were subjected to the same level of ischemia.

## Discussion

Coronary artery occlusion is a major health concern and the principal cause of death in technologically advanced nations. Coronary artery occlusion is also expected to be the main cause of death in the world by the year 2020 (Murray and Lopez 1997). Coronary artery disease is the principal cause of coronary artery occlusion. Importantly, individuals living with SCI have an earlier onset of CAD. In fact, CAD is responsible for nearly 20% of deaths in individuals living with SCI and CAD is expected to become more impactful as the life expectancy of individuals living with SCI increases. Autonomic dysfunction, relative physical inactivity, obesity, hyperlipidemia, insulin resistance, diabetes, and chronic inflammation contribute to CAD in individuals living with SCI (Lee et al. 2005; McKinley et al. 2006).

Importantly, the danger of an untoward cardiac incident is related to the severity of CAD; suggesting that individuals living with SCI may have a greater risk for myocardial infarction or sudden cardiac death. Thus, the management of CAD for individuals living with SCI is a major health concern and therapeutic goal. Primary prevention programs (including smoking cessation, weight control, increasing physical activity, and cholesterol and/or lipid control) are critically important because prevention is the most cost‐effective and harmless strategy. In fact, it has been suggested that the identification of risks for CAD in individuals living with SCI “may be grossly underestimated in those with SCI, requiring a more aggressive approach to determine the presence of atherosclerotic occlusive vascular disease” (Bauman and Spungen 2008).

In this study, we systematically investigated if thoracic level 5 spinal cord transection (T_5_X; paraplegia) alters the arterial blood pressure response to coronary artery occlusion and if the different arterial pressure responses to coronary artery occlusion between intact and paraplegic mice are mediated by changes in cardiac output, peripheral resistance, or both and whether variations in cardiac output are mediated by variations in heart rate and or stroke volume. Paraplegic mice had an impaired ability to maintain arterial blood pressure during coronary artery occlusion as arterial blood pressure fell to near lethal levels within the first 2 min of occlusion. The lower arterial pressure was mediated by a lower cardiac output as peripheral resistance was elevated in paraplegic mice. The lower cardiac output was mediated by a reduced heart rate and stroke volume. These results indicate that in T_5_X mice, the arterial blood pressure response to coronary artery occlusion is hemodynamically mediated primarily by cardiac output which is determined by heart rate and stroke volume. Understanding the response to myocardial ischemia in paraplegic mice has the potential to positively impact individuals and families living with spinal cord injury.

The impaired ability to maintain hemodynamic stability may be related to a failure to activate sympathetic outflow below thoracic level 5. This may be due, in part, to the observation that sympathetic preganglionic neurons (SPNs) below the level of the injury undergo morphological changes after SCI. Krassioukov and Weaver documented in rats that SPNs caudal to the lesion lose dendrites and decrease soma size 1 week after the injury; however, the dendritic arbor of SPNs are re‐established 1 month after the injury (Krassioukov and Weaver 1996). There was also a significant decrease in soma size of SPNs below the level of the injury in an individual who survived for 2 weeks following a SCI at T5 (Krassioukov et al. 1999). However, the soma size from SPNs below the level of the injury was not different from SPNs above the level of the injury from a person who survived 23 years after a SCI at T3. Taken together, SPNs in spinal segments caudal to the lesion appear to undergo a significant decrease in size 2 weeks after SCI; however, the atrophy observed at 2 weeks appears transient as there is a re‐establishment of normal SPN dendritic arbor and soma size within 4 weeks. (Krassioukov et al. 1999). The initial atrophy of SPNs is thought to occur as a result of the loss of descending inputs from medullary neurons (Weaver and Polosa 1994). The initial atrophy of SPNs may contribute to the condition of “spinal shock” where there is a flaccid paralysis, with urinary bladder and bowel atonia, and profound reductions of sympathetic activity. Together, the loss of excitatory drive from medullary centers and atrophy of SPNs likely contribute to reduced sympathetic activity below the level of the injury during the first 2 weeks of a SCI (Teasell et al. 2000).

Furthermore, myocardial ischemia produces metabolites that evoke a powerful sympathetic reflex that increases arterial pressure, heart rate, ST‐segment, and double product (Lujan et al. 2011). This cardiac spinal reflex is analogous to the muscle metaboreflex observed in man, rat, and other mammals (Alam and Smirk 1937; Collins and DiCarlo 1993). Transection of the dorsal roots disrupts this excitatory sympathetic reflex and reduces heart rate and arterial pressure during myocardial ischemia (Lujan et al. 2011). In addition, cardiac output and arterial pressure are reduced during myocardial ischemia which initiates a dramatic arterial baroreflex‐mediated increase in sympathetic outflow and decrease in parasympathetic outflow to the heart as well as an increased sympathetic outflow to the vasculature. As noted, individuals with SCI at thoracic level 5 have a functional loss of the cardiac spinal reflex and the arterial baroreceptor reflex *below* the level of the injury and thus fail to excite sympathetic preganglionic neurons below T_5_. This failure to excite sympathetic preganglionic neurons below the level of the injury likely contributes to the impaired ability to maintain arterial blood pressure and cardiac output during coronary artery occlusion.

The failure to excite sympathetic preganglionic neurons below the level of the injury and reduced activity of the skeletal muscle pump likely results in a profound reduction in end‐diastolic volume and stroke volume, secondary to a reduction in venous return (Lujan and DiCarlo 2014b). This is suggested because reduced activity of the skeletal muscle pump combined with impaired autonomic control of the circulation below the level of the injury can severely impact venous return and reduce cardiac output. In addition, venous pooling can lead to a hypokinetic circulation (Glaser 1985; Glaser and Davis 1989). Moreover, therapeutic techniques that enhance venous return increase stroke volume and cardiac output for individuals living with SCI (Hopman et al. 1992, 1993; Pitetti et al. 1994; Kerk et al. 1995).

In addition, the loss of supra‐spinal control of sympathetic activity to the vasculature innervated below the level of the spinal cord injury results in hypotension. This hypotension initiates a dramatic arterial baroreflex‐mediated increase in sympathetic outflow and decrease in parasympathetic outflow to the heart (Lujan et al. 2012b). High levels of sympathetic outflow and reduced parasympathetic outflow following SCI induce calcium overload (Sharov and Galakhin 1984), left ventricular dysfunction, cardiac injury, and ST‐segment elevation (Morita et al. 2010). These responses likely contribute to the lower stroke volume in paraplegic mice (Lujan and DiCarlo 2014b).

Loss of supraspinal control of SPNs below the level of the injury would be expected to reduce systemic peripheral resistance and prevent vascular resistance from increasing during coronary artery occlusion (West et al. 2013). However, vascular resistance of the legs is often increased in individuals living with SCI (Hopman et al. 2002; Kooijman et al. 2003; Groothuis et al. 2005; Thijssen et al. 2007). Furthermore, individuals living with thoracic SCI have an increase in leg vascular resistance during head‐up tilt that is comparable with that in able‐bodied individuals (Grimm et al. 1997; Raymond et al. 1999; Groothuis et al. 2005). The mechanism(s) most often defined for mediating the elevated vascular resistance in individuals living with SCI are compensatory alterations in other vasoconstrictor pathways including angiotensin II (Krum et al. 1992; Groothuis et al. 2010) and endothelin I (Thijssen et al. 2007) as well as an enhanced sensitivity to norepinephrine (Mathias et al. 1976; Krum et al. 1992; Arnold et al. 1995).

Myocardial ischemia and reperfusion protocols cannot be tested in man. Accordingly, appropriate complex, conscious animal models are essential. In this context, we recorded the response to myocardial ischemia in conscious, chronically instrumented intact and paraplegic mice. It should be considered that the hemodynamic response to coronary artery occlusion is potentially different between rats and mice (Lujan et al. 2012a; Lujan and DiCarlo 2013a,b, 2014a,c). Specifically, we preformed virtually identical procedures in conscious intact and paraplegic rats and every rat developed sustained ventricular tachycardia within 10 min of coronary artery occlusion (Lujan and DiCarlo 2007; Lujan et al. 2009, 2010). Paraplegic mice however did not develop sustained ventricular arrhythmia as the paraplegic mice tolerated the occlusion for <2 min. It is important to note however that these paraplegic mice were studied 2 weeks post SCI while the paraplegic rats were studied 4 weeks post SCI (Lujan and DiCarlo 2007; Lujan et al. 2009, 2010). Understanding the differences between species and/or duration of injury may advance the concepts and ideas that drive therapeutic intervention to improve the quality of life for individuals and families living with SCI.

## Summary and Conclusion

Coronary artery occlusion is a major health concern and a principle cause of death in the world (Murray and Lopez 1997). Autonomic dysfunction, relative inactivity, and adverse changes in muscle mass and adiposity lead to metabolic abnormalities that accelerate the development of CAD; and thus, the potential for myocardial infarction or sudden cardiac death in individuals living with SCI. Accordingly, understanding the response to myocardial ischemia in models of SCI has the potential to be of major importance for advancing the concepts and methods that drive cardiovascular therapies.

Paraplegic mice had an impaired ability to maintain arterial blood pressure during coronary artery occlusion. The lower arterial blood pressure was mediated by a lower cardiac output as peripheral resistance was elevated in paraplegic mice. The lower cardiac output was mediated by a reduced heart rate and stroke volume. These results indicate that in paraplegic mice, the arterial blood pressure response to coronary artery occlusion is hemodynamically mediated primarily by cardiac output which is determined by heart rate and stroke volume.

It should be noted that there are significant differences between mice and man that could profoundly influence the responses. However, studies conducted at many levels, from molecules to man, are critical for finding solutions for individuals with SCI (Lujan et al. 2012a). Accordingly, an extensive array of investigations, rather than a solitary model is required. Accordingly, the conscious mouse provides an additional tool for understanding cardiovascular responses to coronary artery occlusion in models of SCI.

## Conflict of Interest

No conflicts of interest, financial or otherwise, are declared by the author(s).

## References

[phy213214-bib-0001] Alam, M. , and F. H. Smirk . 1937 Observations in man upon a blood pressure raising reflex arising from the voluntary muscles. J. Physiol. 89:372–383. 16994867 10.1113/jphysiol.1937.sp003485 PMC1395054

[phy213214-bib-0002] Arnold, J. M. , Q. P. Feng , G. A. Delaney , and R. W. Teasell . 1995 Autonomic dysreflexia in tetraplegic patients: evidence for alpha‐adrenoceptor hyper‐responsiveness. Clin. Auton. Res. 5:267–270. 8563459 10.1007/BF01818891

[phy213214-bib-0003] Baker, D. G. , H. M. Coleridge , J. C. Coleridge , and T. Nerdrum . 1980 Search for a cardiac nociceptor: stimulation by bradykinin of sympathetic afferent nerve endings in the heart of the cat. J. Physiol. 306:519–536. 7463375 10.1113/jphysiol.1980.sp013412 PMC1283021

[phy213214-bib-0004] Bauman, W. , and A. Spungen . 2007 Risk assessment for coronary heart disease in a veteran population with spinal cord injury. Top. Spinal. Cord. Inj. Rehab. 12:35–53.

[phy213214-bib-0005] Bauman, W. A. , and A. M. Spungen . 2008 Coronary heart disease in individuals with spinal cord injury: assessment of risk factors. Spinal Cord 46:466–476. 18180789 10.1038/sj.sc.3102161

[phy213214-bib-0006] Bauman, W. A. , A. M. Spungen , Y. G. Zhong , J. L. Rothstein , C. Petry , and S. K. Gordon . 1992 Depressed serum high density lipoprotein cholesterol levels in veterans with spinal cord injury. Paraplegia 30:697–703. 1448297 10.1038/sc.1992.136

[phy213214-bib-0007] Bauman, W. A. , M. Raza , Z. Chayes , and J. Machac . 1993 Tomographic thallium‐201 myocardial perfusion imaging after intravenous dipyridamole in asymptomatic subjects with quadriplegia. Arch. Phys. Med. Rehabil. 74:740–744. 8328897 10.1016/0003-9993(93)90036-a

[phy213214-bib-0008] Bauman, W. A. , M. Raza , A. M. Spungen , and J. Machac . 1994 Cardiac stress testing with thallium‐201 imaging reveals silent ischemia in individuals with paraplegia. Arch. Phys. Med. Rehabil. 75:946–950. 8085927

[phy213214-bib-0009] Bauman, W. A. , R. H. Adkins , A. M. Spungen , R. Herbert , C. Schechter , D. Smith , et al. 1999 Is immobilization associated with an abnormal lipoprotein profile? Observations from a diverse cohort. Spinal Cord 37:485–493. 10438115 10.1038/sj.sc.3100862

[phy213214-bib-0010] Bohl, S. , D. J. Medway , J. Schulz‐Menger , J. E. Schneider , S. Neubauer , and C. A. Lygate . 2009 Refined approach for quantification of in vivo ischemia‐reperfusion injury in the mouse heart. Am. J. Physiol. Heart Circ. Physiol. 297:H2054–H2058. 19820193 10.1152/ajpheart.00836.2009 PMC2793132

[phy213214-bib-0011] Cardus, D. , F. Ribas‐Cardus , and W. G. McTaggart . 1992 Lipid profiles in spinal cord injury. Paraplegia 30:775–782. 1484728 10.1038/sc.1992.149

[phy213214-bib-0012] Collins, H. L. , and S. E. DiCarlo . 1993 Cardiac afferents attenuate the muscle metaboreflex in the rat. J. Appl. Physiol. 75:114–120. 8376257 10.1152/jappl.1993.75.1.114

[phy213214-bib-0013] Cragg, J. J. , V. K. Noonan , A. Krassioukov , and J. Borisoff . 2013 Cardiovascular disease and spinal cord injury: results from a national population health survey. Neurology 81:723–728. 23884034 10.1212/WNL.0b013e3182a1aa68 PMC3776463

[phy213214-bib-0014] Curtis, M. J. , J. C. Hancox , A. Farkas , C. L. Wainwright , C. L. Stables , D. A. Saint , et al. 2013 The Lambeth Conventions (II): guidelines for the study of animal and human ventricular and supraventricular arrhythmias. Pharmacol. Ther. 139:213–248. 23588158 10.1016/j.pharmthera.2013.04.008

[phy213214-bib-0015] DeVivo, M. J. 1997 Causes and costs of spinal cord injury in the United States. Spinal Cord 35:809–813. 9429259 10.1038/sj.sc.3100501

[phy213214-bib-0016] DeVivo, M. J. , K. J. Black , and S. L. Stover . 1993 Causes of death during the first 12 years after spinal cord injury. Arch. Phys. Med. Rehabil. 74:248–254. 8439250

[phy213214-bib-0017] Fleg, J. L. , G. Gerstenblith , A. B. Zonderman , L. C. Becker , M. L. Weisfeldt , P. T. Jr Costa , et al. 1990 Prevalence and prognostic significance of exercise‐induced silent myocardial ischemia detected by thallium scintigraphy and electrocardiography in asymptomatic volunteers. Circulation 81:428–436. 2297853 10.1161/01.cir.81.2.428

[phy213214-bib-0018] Frankel, H. L. , J. R. Coll , S. W. Charlifue , G. G. Whiteneck , B. P. Gardner , M. A. Jamous , et al. 1998 Long‐term survival in spinal cord injury: a fifty year investigation. Spinal Cord 36:266–274. 9589527 10.1038/sj.sc.3100638

[phy213214-bib-0019] Fu, L. W. , and J. C. Longhurst . 2002 Activated platelets contribute to stimulation of cardiac afferents during ischaemia in cats: role of 5‐HT(3) receptors. J. Physiol. 544:897–912. 12411532 10.1113/jphysiol.2002.023374 PMC2290632

[phy213214-bib-0020] Fu, L. W. , and J. C. Longhurst . 2005 Interactions between histamine and bradykinin in stimulation of ischaemically sensitive cardiac afferents in felines. J. Physiol. 565:1007–1017. 15774520 10.1113/jphysiol.2005.084004 PMC1464556

[phy213214-bib-0021] Fu, L. W. , W. Schunack , and J. C. Longhurst . 2005 Histamine contributes to ischemia‐related activation of cardiac spinal afferents: role of H1 receptors and PKC. J. Neurophysiol. 93:713–722. 15653785 10.1152/jn.00528.2004

[phy213214-bib-0022] Glaser, R. M. 1985 Exercise and locomotion for the spinal cord injured. Exerc. Sport Sci. Rev. 13:263–303. 3891369

[phy213214-bib-0023] Glaser, R. , and G. Davis **.** 1989 Wheelchair‐dependent individuals *in* Exercise in modern medicine. Williams & Wilkins, Baltimore.

[phy213214-bib-0024] Grimm, D. R. , R. E. De Meersman , P. L. Almenoff , A. M. Spungen , and W. A. Bauman . 1997 Sympathovagal balance of the heart in subjects with spinal cord injury. Am. J. Physiol. 272:H835–H842. 9124446 10.1152/ajpheart.1997.272.2.H835

[phy213214-bib-0025] Groah, S. L. , D. Weitzenkamp , P. Sett , B. Soni , and G. Savic . 2001 The relationship between neurological level of injury and symptomatic cardiovascular disease risk in the aging spinal injured. Spinal Cord 39:310–317. 11438852 10.1038/sj.sc.3101162

[phy213214-bib-0026] Groothuis, J. T. , C. R. Boot , S. Houtman , H. van Langen , and M. T. Hopman . 2005 Leg vascular resistance increases during head‐up tilt in paraplegics. Eur. J. Appl. Physiol. 94:408–414. 15843958 10.1007/s00421-005-1340-5

[phy213214-bib-0027] Groothuis, J. T. , D. H. Thijssen , G. A. Rongen , J. Deinum , A. H. Danser , A. C. Geurts , et al. 2010 Angiotensin II contributes to the increased baseline leg vascular resistance in spinal cord‐injured individuals. J. Hypertens. 28:2094–2101. 20577118 10.1097/HJH.0b013e32833cd2f4

[phy213214-bib-0028] Haywood, J. R. , R. A. Shaffer , C. Fastenow , G. D. Flink , and M. J. Brody . 1981 Regional blood flow measurements with pulsed Doppler flowmeter in conscious rat. Am. J. Physiol. Heart Circ. Physiol. 241:H273–H278. 10.1152/ajpheart.1981.241.2.H273 6455924

[phy213214-bib-0029] Hopman, M. T. , B. Oeseburg , and R. A. Binkhorst . 1992 The effect of an anti‐G suit on cardiovascular responses to exercise in persons with paraplegia. Med. Sci. Sports Exerc. 24:984–990. 1406199

[phy213214-bib-0030] Hopman, M. T. , I. C. Kamerbeek , M. Pistorius , and R. A. Binkhorst . 1993 The effect of an anti‐G suit on the maximal performance of individuals with paraplegia. Int. J. Sports Med. 14:357–361. 8244600 10.1055/s-2007-1021192

[phy213214-bib-0031] Hopman, MT , JT Groothuis , M Flendrie , KH Gerrits , and S Houtman . 2002 Increased vascular resistance in paralyzed legs after spinal cord injury is reversible by training. J. App. Physiol. (Bethesda, Md: 1985) 93: 1966–1972. 10.1152/japplphysiol.00897.2001 12433934

[phy213214-bib-0032] Inskip, J. , W. Plunet , L. Ramer , J. B. Ramsey , A. Yung , P. Kozlowski , et al. 2010 Cardiometabolic risk factors in experimental spinal cord injury. J. Neurotrauma 27:275–285. 19772460 10.1089/neu.2009.1064

[phy213214-bib-0033] Kerk, J. K. , P. S. Clifford , A. C. Snyder , T. E. Prieto , K. P. O'Hagan , P. K. Schot , et al. 1995 Effect of an abdominal binder during wheelchair exercise. Med. Sci. Sports Exerc. 27:913–919. 7658955

[phy213214-bib-0034] Kessler, K. M. , I. Pina , B. Green , B. Burnett , M. Laighold , M. Bilsker , et al. 1986 Cardiovascular findings in quadriplegic and paraplegic patients and in normal subjects. Am. J. Cardiol. 58:525–530. 3751915 10.1016/0002-9149(86)90027-5

[phy213214-bib-0035] Kooijman, M. , G. A. Rongen , P. Smits , and M. T. Hopman . 2003 Preserved alpha‐adrenergic tone in the leg vascular bed of spinal cord‐injured individuals. Circulation 108:2361–2367. 14557353 10.1161/01.CIR.0000096480.55857.3C

[phy213214-bib-0036] Krassioukov, A. V. , and L. C. Weaver . 1996 Morphological changes in sympathetic preganglionic neurons after spinal cord injury in rats. Neuroscience 70:211–225. 8848126 10.1016/0306-4522(95)00294-s

[phy213214-bib-0037] Krassioukov, A. V. , R. P. Bunge , W. R. Pucket , and M. A. Bygrave . 1999 The changes in human spinal sympathetic preganglionic neurons after spinal cord injury. Spinal Cord 37:6–13. 10025688 10.1038/sj.sc.3100718

[phy213214-bib-0038] Krum, H. , W. J. Louis , D. J. Brown , and L. G. Howes . 1992 Pressor dose responses and baroreflex sensitivity in quadriplegic spinal cord injury patients. J. Hypertens. 10:245–250. 1315821 10.1097/00004872-199203000-00007

[phy213214-bib-0039] Kurtz, T. W. , H. L. Lujan , and S. E. DiCarlo . 2014 The 24 h pattern of arterial pressure in mice is determined mainly by heart rate‐driven variation in cardiac output. Physiol. Rep. 2. 10.14814/phy2.12223 PMC4255824 25428952

[phy213214-bib-0040] Lee, M. Y. , J. Myers , A. Hayes , S. Madan , V. F. Froelicher , I. Perkash , et al. 2005 C‐reactive protein, metabolic syndrome, and insulin resistance in individuals with spinal cord injury. J. Spinal Cord Med. 28:20–25. 15832900 10.1080/10790268.2005.11753794

[phy213214-bib-0041] Lee, C. S. , Y. H. Lu , S. T. Lee , C. C. Lin , and H. J. Ding . 2006 Evaluating the prevalence of silent coronary artery disease in asymptomatic patients with spinal cord injury. Int. Heart J. 47:325–330. 16823238 10.1536/ihj.47.325

[phy213214-bib-0042] Longhurst, J. C. , A. L. S. Tjen , and L. W. Fu . 2001 Cardiac sympathetic afferent activation provoked by myocardial ischemia and reperfusion. Mechanisms and reflexes. Ann. N. Y. Acad. Sci. 940:74–95. 11458709 10.1111/j.1749-6632.2001.tb03668.x

[phy213214-bib-0043] Lujan, H. L. , and S. E. DiCarlo . 2007 T5 spinal cord transection increases susceptibility to reperfusion‐induced ventricular tachycardia by enhancing sympathetic activity in conscious rats. Am. J. Physiol. Heart Circ. Physiol. 293:H3333–H3339. 17933964 10.1152/ajpheart.01019.2007

[phy213214-bib-0044] Lujan, H. L. , and S. E. DiCarlo . 2013a Cardiac output, at rest and during exercise, before and during myocardial ischemia, reperfusion, and infarction in conscious mice. Am. J. Physiol. Regul. Integr. Comp. Physiol. 304:R286–R295. 23302959 10.1152/ajpregu.00517.2012 PMC3567356

[phy213214-bib-0045] Lujan, H. L. , and S. E. DiCarlo . 2013b Mimicking the endogenous current of injury improves post‐infarct cardiac remodeling. Med. Hypotheses 81:521–523. 23871584 10.1016/j.mehy.2013.06.022

[phy213214-bib-0046] Lujan, H. L. , and S. E. DiCarlo . 2014a Cardiac electrophysiology and the susceptibility to sustained ventricular tachycardia in intact, conscious mice. Am. J. Physiol. Heart Circ. Physiol. 306:H1213–H1221. 24561859 10.1152/ajpheart.00780.2013 PMC3989750

[phy213214-bib-0047] Lujan, H. L. , and S. E. DiCarlo . 2014b Increasing venous return as a strategy to prevent or reverse cardiac dysfunction following spinal cord injury. J. Physiol. 592:1727–1728. 24737897 10.1113/jphysiol.2014.272666 PMC4001746

[phy213214-bib-0048] Lujan, H. L. , and S. E. DiCarlo . 2014c Reperfusion‐induced sustained ventricular tachycardia, leading to ventricular fibrillation, in chronically instrumented, intact, conscious mice. Physiol. Rep. 2. 10.14814/phy2.12057 PMC4208649 24973331

[phy213214-bib-0049] Lujan, H. L. , Y. Chen , and S. E. DiCarlo . 2009 Paraplegia Increased Cardiac NGF Content, Sympathetic Tonus and the Susceptibility to Ischemia‐Induced Ventricular Tachycardia in Conscious Rats. Am. J. Physiol. Heart Circ. Physiol. 296:H1364–H1372. 19286942 10.1152/ajpheart.01286.2008 PMC2685353

[phy213214-bib-0050] Lujan, H. L. , G. Palani , L. Zhang , and S. E. DiCarlo . 2010 Targeted Ablation of Cardiac Sympathetic Neurons Reduces the Susceptibility to Ischemia‐Induced Sustained Ventricular Tachycardia in Conscious Rats. Am. J. Physiol. Heart Circ. Physiol. 298:H1330–H1339. 20173045 10.1152/ajpheart.00955.2009 PMC2867448

[phy213214-bib-0051] Lujan, H. L. , S. Krishnan , and S. E. DiCarlo . 2011 Cardiac spinal deafferentation reduces the susceptibility to sustained ventricular tachycardia in conscious rats. Am. J. Physiol. Regul. Integr. Comp. Physiol. 301:R775–R782. 21677267 10.1152/ajpregu.00140.2011 PMC3174758

[phy213214-bib-0052] Lujan, H. L. , H. Janbaih , H. Z. Feng , J. P. Jin , and S. E. DiCarlo . 2012a Myocardial ischemia, reperfusion, and infarction in chronically instrumented, intact, conscious, and unrestrained mice. Am. J. Physiol. Regul. Integr. Comp. Physiol. 302:R1384–R1400. 22538514 10.1152/ajpregu.00095.2012 PMC3378340

[phy213214-bib-0053] Lujan, H. L. , H. Janbaih , H. Z. Feng , J. P. Jin , and S. E. DiCarlo . 2012b Ventricular function during exercise in mice and rats. Am. J. Physiol. Regul. Integr. Comp. Physiol. 302:R68–R74. 22012697 10.1152/ajpregu.00340.2011 PMC3349383

[phy213214-bib-0054] Malliani, A. , F. Lombardi , and M. Pagani . 1981 Functions of afferents in cardiovascular sympathetic nerves. J. Auton. Nerv. Syst. 3:231–236. 7276432 10.1016/0165-1838(81)90065-5

[phy213214-bib-0055] Mathias, C. J. , H. L. Frankel , N. J. Christensen , and J. N. K. Spalding . 1976 Enhanced pressor response to noradrenaline in patients with cervical spinal cord transection. Brain 99:757–770. 1030656 10.1093/brain/99.4.757

[phy213214-bib-0056] McKinley, W. **,** S Garstang , J Wieting , F Talavera , K Allen , and D Campagnolo . 2006 Cardiovascular Concerns in Spinal Cord Injury. eMedicine eMedicine Specialties/Physical Medicine and Rehabilitation/Spinal Cord Injury Available at: http://emedicine.medscape.com/article/321771-overview (accessed 29 December 2016)

[phy213214-bib-0057] Meller, S. T. , and G. F. Gebhart . 1992 A critical review of the afferent pathways and the potential chemical mediators involved in cardiac pain. Neuroscience 48:501–524. 1351270 10.1016/0306-4522(92)90398-l

[phy213214-bib-0058] Morita, S. , S. Inokuchi , T. Yamagiwa , H. Aoki , Y. Nakagawa , and I. Yamamoto . 2010 Tako‐tsubo‐like left ventricular dysfunction with ST‐segment elevation after central spinal cord injury: a case report. J. Emerg. Med. 39:301–304. 18597972 10.1016/j.jemermed.2007.10.086

[phy213214-bib-0059] Murray, C. J. L. , and A. D. Lopez . 1997 Alternative projection of mortality and disability by cause 1990‐2020: Global burden of disease study. Lancet 349:1498–1504. 9167458 10.1016/S0140-6736(96)07492-2

[phy213214-bib-0060] Orakzai, S. H. , R. H. Orakzai , N. Ahmadi , N. Agrawal , W. A. Bauman , F. Yee , et al. 2007 Measurement of coronary artery calcification by electron beam computerized tomography in persons with chronic spinal cord injury: evidence for increased atherosclerotic burden. Spinal Cord 45:775–779. 17339887 10.1038/sj.sc.3102045

[phy213214-bib-0061] Pan, H. L. , J. C. Longhurst , J. C. Eisenach , and S. R. Chen . 1999 Role of protons in activation of cardiac sympathetic C‐fibre afferents during ischaemia in cats. J. Physiol. 518(Pt 3):857–866. 10420020 10.1111/j.1469-7793.1999.0857p.x PMC2269450

[phy213214-bib-0062] Pitetti, K. H. , P. J. Barrett , K. D. Campbell , and D. E. Malzahn . 1994 The effect of lower body positive pressure on the exercise capacity of individuals with spinal cord injury. Med. Sci. Sports Exerc. 26:463–468. 8201903

[phy213214-bib-0063] Raymond, J. , G. M. Davis , G. Bryant , and J. Clarke . 1999 Cardiovascular responses to an orthostatic challenge and electrical‐stimulation‐induced leg muscle contractions in individuals with paraplegia. Eur. J. Appl. Physiol. Occup. Physiol. 80:205–212. 10453922 10.1007/s004210050583

[phy213214-bib-0064] Sharov, V. G. , and K. A. Galakhin . 1984 Myocardial changes after spinal cord injuries in humans and experimental animals. Arkh. Patol. 46:17–20. 6466137

[phy213214-bib-0065] Strack, A. M. , W. B. Sawyer , L. M. Marubio , and A. D. Loewy . 1988 Spinal origin of sympathetic preganglionic neurons in the rat. Brain Res. 455:187–191. 3416186 10.1016/0006-8993(88)90132-1

[phy213214-bib-0066] Swoap, S. J. , J. M. Overton , and G. Garber . 2004 Effect of ambient temperature on cardiovascular parameters in rats and mice: a comparative approach. Am. J. Physiol. Regul. Integr. Comp. Physiol. 287:R391–R396. 15087284 10.1152/ajpregu.00731.2003

[phy213214-bib-0067] Teasell, R. W. , J. M. O. Arnold , A. Krassioukov , and G. A. Delaney . 2000 Cardiovascular consequences of loss of supraspinal control of the sympathetic nervous system after spinal cord injury. Arch. Phys. Med. Rehabil. 81:506–516. 10768544 10.1053/mr.2000.3848

[phy213214-bib-0068] Thijssen, D. H. , R. Ellenkamp , M. Kooijman , P. Pickkers , G. A. Rongen , M. T. Hopman , et al. 2007 A causal role for endothelin‐1 in the vascular adaptation to skeletal muscle deconditioning in spinal cord injury. Arterioscler. Thromb. Vasc. Biol. 27:325–331. 17122448 10.1161/01.ATV.0000253502.83167.31

[phy213214-bib-0069] Tjen, A. L. , L. W. Fu , and J. C. Longhurst . 2002 Xanthine oxidase, but not neutrophils, contributes to activation of cardiac sympathetic afferents during myocardial ischaemia in cats. J. Physiol. 543:327–336. 12181303 10.1113/jphysiol.2001.013482 PMC2290482

[phy213214-bib-0070] Uchida, Y. , and S. Murao . 1974 Excitation of afferent cardiac sympathetic nerve fibers during coronary occlusion. Am. J. Physiol. 226:1094–1099. 4824862 10.1152/ajplegacy.1974.226.5.1094

[phy213214-bib-0071] Weaver, L. , and C. Polosa . 1994 Spinal cord circuits providing control of sympathetic preganglionic neurons in Jordan D., ed. The autonomic nervous system control of autonomic functions. Harwood Academic Publishers, London.

[phy213214-bib-0072] West, C. R. , P. Mills , and A. V. Krassioukov . 2012 Influence of the neurological level of spinal cord injury on cardiovascular outcomes in humans: a meta‐analysis. Spinal Cord 50:484–492. 22391687 10.1038/sc.2012.17

[phy213214-bib-0073] West, C. R. , A. Alyahya , I. Laher , and A. Krassioukov . 2013 Peripheral vascular function in spinal cord injury: a systematic review. Spinal Cord 51:10–19. 23184028 10.1038/sc.2012.136

[phy213214-bib-0074] Whiteneck, G. G. , S. W. Charlifue , H. L. Frankel , M. H. Fraser , B. P. Gardner , K. A. Gerhart , et al. 1992 Mortality, morbidity, and psychosocial outcomes of persons spinal cord injured more than 20 years ago. Paraplegia 30:617–630. 1408338 10.1038/sc.1992.124

